# Liposarcoma of the retropharyngeal space with rapidly worsening dyspnea: A case report and review of the literature

**DOI:** 10.3892/ol.2013.1310

**Published:** 2013-04-17

**Authors:** JIAN-GUO HE, HUA JIANG, BEI-BEI YANG, PENG-FANG LIN

**Affiliations:** Department of Otolaryngology, Second Affiliated Hospital, School of Medicine, Zhejiang University, Hangzhou, Zhejiang 310009, P.R. China

**Keywords:** liposarcoma, retropharyngeal space, dyspnea

## Abstract

Liposarcomas represent a significant proportion of soft-tissue sarcomas. However, their occurrence in the head and neck is infrequent and they are exceedingly rare in the retropharyngeal space. The present study reports the case of a 58-year-old patient with retropharyngeal liposarcoma. Uniquely, the patient presented with rapidly worsening dyspnea. The diagnosis of liposarcoma was established following retropharyngeal tumor excision, although biopsies were performed twice. Adjuvant radiotherapy was refused by the patient. However, during the post-operative follow-up period, no sign of either local tumor recurrence or distant metastasis was observed. Previously reported cases were also reviewed to analyze the diagnosis, treatment and prognosis of this disease.

## Introduction

Liposarcoma is the most common type of soft-tissue sarcoma. However, head and neck liposarcomas are unusual, accounting for only 1.8–6.3% of cases (1). Furthermore, cases of liposarcoma in the retropharyngeal space are extremely rare. To the best of our knowledge, only five cases have been reported previously (1–5). Unlike previous cases, the present study reports a highly unusual case of retropharyngeal liposarcoma with rapidly worsening dyspnea and total dysphagia following an accident and subsequent surgery. Due to the difficulties of biopsy, radiological examination and pathohistological examination, a retropharyngeal well-differentiated liposarcoma may be easily misdiagnosed as a lipoma. The retropharyngeal space is extremely close to vital neurovascular structures and the extent of any surgical excision is restricted to avoid severe complications. These difficulties may affect the prognosis of patients with retropharyngeal liposarcoma. In the present study, the diagnosis, treatment and prognosis of this disease is discussed by analyzing the present case and by reviewing previously reported cases. This study was approved by the ethics committee of the Second Affiliated Hospital, School of Medicine, Zhejiang University. Written informed consent was obtained from the patient.

## Case report

**Figure 1:**
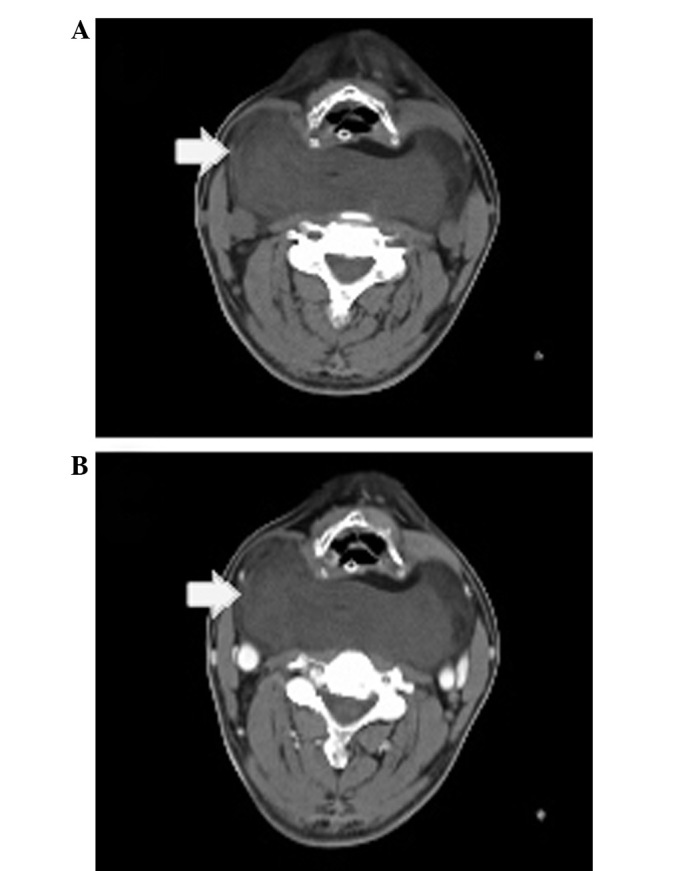
Computed tomography (CT) scans. (A) Retropharyngeal space occupied by an extremely large mass (arrow) extending to the sides of the neck. (B) The mass (arrow) was not enhanced following contrast agent administration.

A 58-year-old male patient presented with rapidly worsening dyspnea and total dysphagia occurring within several hours. Three years earlier the patient had noted bilateral neck swelling. The swelling grew slowly and one year later the patient developed mild dysphagia. As the patient was unaware of the potential severity of the symptom, a prompt examination and treatment were not provided. This symptom did not markedly progress until the occurrence of a traffic accident. Due to this accident, the patient underwent tibiofibular fracture surgery at a local hospital. Post-operatively, the patient recovered well and ate as usual. However, three days subsequent to the surgery, the patient suddenly developed rapidly worsening dyspnea and total dysphagia. Attempts at intubation failed, so a tracheostomy was performed under local anesthesia. The patient was then referred to the Department of Otolaryngology (Second Affiliated Hospital, Zhejiang University, Hangzhou, China). A physical examination revealed an extremely large, soft, non-tender mass measuring ∼11×10×8 cm, involving the bilateral neck. A laryngoscopy showed a retropharyngeal mass that was reducing the space of the pharynx. A computed tomography (CT) scan of the neck revealed a large, well-circumscribed, fatty, dense mass measuring 11×11×9 cm, which extended from the retropharyngeal space to the sides of the neck and from the level of the hyoid bone to the superior margin of the mediastinum. The mass displaced the trachea and larynx anteriorly and the carotid arteries laterally. The mass was not enhanced following contrast agent administration (Fig. 1A and B). Due to the internal fixation of the leg, magnetic resonance imaging (MRI) was not an option. In addition, a CT scan of the chest and abdomen was performed and no similar mass was observed.

**Figure 2:**
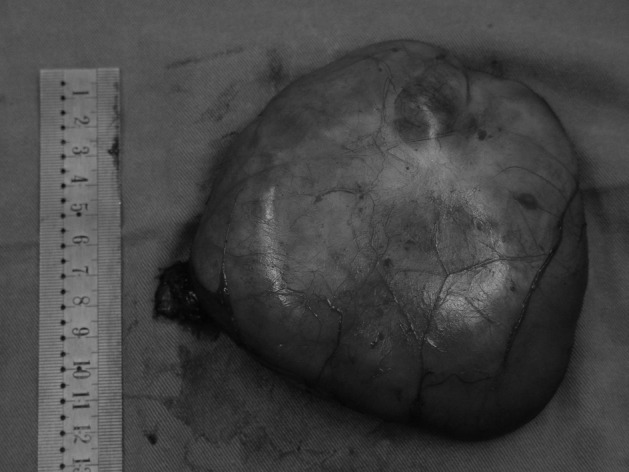
Resected tumor encapsulated and measured as 12.5×12×7 cm.

**Figure 3:**
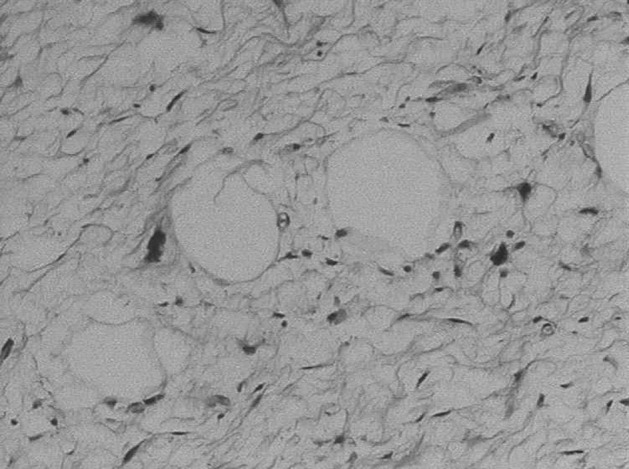
Histology showing well-differentiated liposarcoma (hematoxylin-eosin staining, original magnification, ×100).

An ultrasound-guided core biopsy of the mass revealed histological components of fibrous, vascular and fatty tissues. An incisional biopsy of the lesion was then performed with the patient under local anesthesia. The histology indicated a vascular fibrous lipoma. Subsequently, a surgical excision of the retropharyngeal mass was performed under general anesthesia using an H-shaped incision. The tumor was well-encapsulated and adhered to the posterior pharyngeal wall. The tumor was subsequently resected completely and measured as weighing 401 g (Fig. 2). The posterior pharyngeal and esophageal walls were completely preserved. Microscopically, the lesion had components of mature adipocytes and lipoblasts with nuclear atypia (Fig. 3). A diagnosis of a well-differentiated liposarcoma was confirmed. The suggested adjuvant radiotherapy was not accepted by the patient.

During the post-operative course, the patient developed vocal hoarseness. A laryngoscopy revealed right-sided vocal fold weakness, which the patient recovered from one month later. The patient was decannulated and the nasogastric tube was removed. Upon follow-up at 20 months, there were no signs of either local tumor recurrence or distant metastasis.

## Discussion

The retropharyngeal space is the potential space lying between the prevertebral fascia posteriorly and the buccopharyngeal membrane covering the constrictor muscles anteriorly. It extends from the skull base to the mediastinum. The retropharyngeal space is separated from the parapharyngeal space by a thin fascial layer and closed by the internal jugular vein, common carotid artery and vagus nerve. Liposarcomas usually present as painless enlarging masses and when they arise in the retropharyngeal space they are difficult to detect early and are usually discovered incidentally. Patients with a retropharyngeal mass usually present with dysphagia and foreign body sensation and when these symptoms are not as clear the patients may become habituated to their symptoms. Physical findings include bilateral neck swelling and a reduced anteroposterior diameter of the pharynx, although these do not appear until the tumors have reached a large size (1–5). In the present case, as in all five previously reported cases, prior to the liposarcoma being identified, it had attained an extremely large size and compressed the pharynx, causing dysphagia and ultimately dyspnea. However, unlike previous cases, the present patient presented rapidly worsening dyspnea and total dysphagia following tibiofibular fracture surgery. This indicates that stresses, including trauma or surgery, may stimulate the growth of the tumor. Similarly, stress and surgical intervention have also been demonstrated to promote tumor development in another study (6). However, the mechanism remains unclear. Natural killer cell activity and β-adrenergic receptors may be involved in this process (6).

Lipocytes of various shapes and sizes with nuclear atypia indicate a diagnosis of liposarcoma. The classification proposed by Enzinger and Weiss is widely accepted and has been adopted by the World Health Organization. This classification identifies four subtypes of liposarcoma: well-differentiated, myxoid, dedifferentiated and pleomorphic (7). The histological appearances of these vary from well-differentiated neoplasms with scattered atypical cells to pleomorphic neoplasms resembling high-grade malignant fibrous histiocytomas (7). CT and MRI scans aid in establishing a diagnosis. Murphey *et al* indicated that well-differentiated liposarcomas are frequently diagnosed in CT or MRI scans, with a largely lipomatous mass (>75% of the lesion) and non-lipomatous components in thick septa or focal nodules (8). The identification of a nodular dominant focus (>1 cm in size) of non-lipomatous tissue using CT or MRI suggests a dedifferentiated liposarcoma (8,9). Myxoid liposarcomas have a high water content and pleomorphic liposarcomas are high-grade sarcomatous lesions that typically appear as heterogeneous soft-tissue masses (8). However, a well-differentiated liposarcoma is difficult to distinguish from a lipoma by CT or MRI scans and is commonly misdiagnosed, even with a biopsy. Notably, all reported cases, including the present case, were well-differentiated liposarcoma, although 33.3% were misdiagnosed as lipoma pre-operatively (1–5). In retropharyngeal liposarcomas, due to the deep location and the large tumor, a core biopsy or incisional biopsy usually obtains only a small part of the tumor. This may be one of the reasons behind the frequent misdiagnoses.

The principal management approach for liposarcoma is wide surgical excision. However, in the head and neck, the lesion is usually close to vital neurovascular structures, so the extent of surgical excision is restricted to avoid severe complications and the use of adjuvant radiotherapy is increasing. Certain studies have demonstrated the effect of radiotherapy in reducing the rate of local recurrence of liposarcomas (10). Eeles *et al* observed that adjuvant radiotherapy significantly decreased the rate of local recurrence of head and neck sarcomas (11). Our previous study also showed that adjuvant radiotherapy is important in the management of sarcomas of the parapharyngeal space (12). However, for well-differentiated liposarcomas of the head and neck, certain authors have suggested that wide surgical resection is sufficient (13). The majority of studies have been confined by the small series of patients used to assess the value of adjuvant radiotherapy in the treatment of head and neck liposarcomas (14,15).

Among the various series of retropharyngeal liposarcomas in the literature, only one patient received adjuvant radiotherapy in the initial treatment and the follow-up revealed no evidence of recurrence (2). Among the other five patients with surgery alone, only one patient developed recurrence within a year and received re-excision with adjuvant radiotherapy (1). Six months after re-excision, there were no signs of recurrence (1). Additionally, from these five patients, the follow-up of the present patient was the longest and there have been no signs of either recurrence or metastasis. Although the reported periods of follow-up were not long enough, with two cases with a follow-up of less than one year (2,3), one case with no follow-up reported (4) and the other three, including the present case, reporting a follow-up of between 18 and 20 months (1,5), no clear benefits of adjuvant radiotherapy were observed.

The major prognostic factor for liposarcoma is the histological subtype. Well-differentiated liposarcoma is the most common subtype of all liposarcomas, recurring locally, but rarely metastasizing (16). Patients with this type of liposarcoma have an improved prognosis compared with those with other subtypes (17). Myxoid tumors, similar to the well-differentiated variety, are unlikely to metastasize and have a favorable five-year survival rate. However, these tumors have high local recurrence rates and are more locally invasive (18). The other two subtypes are significantly more aggressive and have worse prognoses (18).

Although all six reported cases of retropharyngeal liposarcoma were of the well-differentiated subtype, certain characteristics of this unusual tumor may affect the prognosis due to its unique localization. Late detection may provide sufficient time for tumor invasion and metastases to occur. Misdiagnosis may lead to the diagnosis of a benign lesion, which may result in a limitation of the extent of surgical excision. In addition, liposarcomas located in deep anatomical sites, including the retroperitoneum and mediastinum, have a relatively unfavorable prognosis (19). This may be due to the difficulties of using adequate surgical margins. Similarly, in the head and neck, patients with facial, scalp and laryngeal tumors have an improved prognosis compared with those with intraoral, pharyngeal and neck tumors (19). The retropharyngeal space is a deep potential space that is extremely close to vital neurovascular structures, so the extent of excision is restricted to avoid severe complications. The difficulties of surgery may also affect the prognosis of patients with retropharyngeal liposarcoma.

In conclusion, although well-differentiated liposarcoma is the most common subtype of liposarcoma and has an improved prognosis, retropharyngeal liposarcoma has a number of unfavorable prognostic factors due to its unique localization. No adequate evidence has demonstrated the value of adjuvant radiotherapy for treating retropharyngeal liposarcomas. However, for liposarcomas, adjuvant radiotherapy has been shown to decrease the rate of local recurrence in a number of literature studies.
